# Superconducting ferecrystals: turbostratically disordered atomic-scale layered (PbSe)_1.14_(NbSe_2_)_*n*_ thin films

**DOI:** 10.1038/srep33457

**Published:** 2016-09-16

**Authors:** Corinna Grosse, Matti B. Alemayehu, Matthias Falmbigl, Anna Mogilatenko, Olivio Chiatti, David C. Johnson, Saskia F. Fischer

**Affiliations:** 1 Novel Materials Group, Humboldt-Universität zu Berlin, 12489 Berlin, Germany; 2 Department of Chemistry, University of Oregon, Eugene, Oregon 97403, United States; 3 Ferdinand-Braun-Institut, Leibniz-Institut für Höchstfrequenztechnik, 12489 Berlin, Germany

## Abstract

Hybrid electronic heterostructure films of semi- and superconducting layers possess very different properties from their bulk counterparts. Here, we demonstrate superconductivity in ferecrystals: turbostratically disordered atomic-scale layered structures of single-, bi- and trilayers of NbSe_2_ separated by PbSe layers. The turbostratic (orientation) disorder between individual layers does not destroy superconductivity. Our method of fabricating artificial sequences of atomic-scale 2D layers, structurally independent of their neighbours in the growth direction, opens up new possibilities of stacking arbitrary numbers of hybrid layers which are not available otherwise, because epitaxial strain is avoided. The observation of superconductivity and systematic *T*_c_ changes with nanostructure make this synthesis approach of particular interest for realizing hybrid systems in the search of 2D superconductivity and the design of novel electronic heterostructures.

Stacked low-dimensional NbSe_2_ layers are topical with respect to collective many-body phenomena in transport, superconductivity and charge density waves (CDWs)^1^,^2^,^3^,^4^,^5^,^6^,^7^,^8^,^9^,^10^. Recently, superconductivity was shown in exfoliated NbSe_2_ single, bi- and trilayers^1^,^11^,^12^,^13^ and strong evidence for two-dimensional (2D) superconductivity was found^13^. However, by exfoliation only small-scale flake-like layers may be produced and atomic-scale layering of hybrid materials is limited due to the challenges involved in sequentially stacking layers. Due to strain, epitaxial growth is limited to few stacking sequences and does not exist for thin extended films. Instead, for ferecrystals a manifold of stacking sequences is possible, because no epitaxial relationship is given for subsequent atomic planes in growth direction^14^.

We have developed a controlled synthesis via the modulated elemental reactants (MER) method^15^,^16^,^17^ to obtain specifically designed stacking sequences. Orientation disorder between the constituents leads to structurally independent layers without structural distortions and strain. However, to date, it is unclear whether and how orientation disorder between successive atomic layers may influence emergent phenomena, such as the normal-to-superconducting transition. Here, we show that (PbSe)_1.14_(NbSe_2_)_*n*_ ferecrystals become superconducting, despite the turbostratic disorder.

In general, ferecrystals are described by the formula [(*MX*)_1+*x*_]_*m*_(*TX*_2_)_*n*_, with *M* = metal, *T* = transition metal and *X* = Se, S or Te, and *m* atomic bilayers of *MX* are alternately stacked with *n TX*_2_ monolayers. Ferecrystals are prepared by self-assembly of designed precursors^14^,^15^,^16^,^17^,^18^. Their synthesis method has been developed only recently. It opens the opportunity to embed 2D NbSe_2_ layers, which are susceptible for oxidation and contamination, in a semiconducting material such as PbSe for devices allowing for oxidation protection as well as for gating applications.

Here, we report the synthesis, the structural and the electrical properties of (PbSe)_1.14_(NbSe_2_)_*n*_ ferecrystals with *n* = 1, 2, 3 in order to study the superconducting transition temperatures. The undistorted and crystallographically independent NbSe_2_ layers in the ferecrystals are essentially buried 2D layers, which is the ultimate geometry of devices that will be constructed from 2D materials. Superconductivity can be used as a sensitive probe of the electronic and vibrational interactions between the constituent layers.

## Results

### The structure of ferecrystals

**Figure 1:**
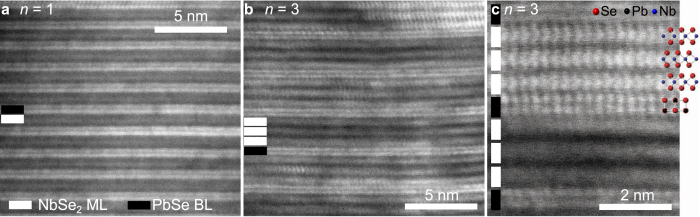
Cross-sectional HAADF-STEM images of (PbSe)_1.14_(NbSe2)_*n*_ ferecrystals. (**a**) The layer structure is shown on the atomic scale for *n* = 1. A NbSe_2_ monolayer (ML) is indicated by a white bar and a PbSe atomic bilayer (BL) is indicated by a black bar. (**b**) HAADF-STEM image of *n* = 3. (**c**) High-resolution HAADF-STEM image of *n* = 3 showing single atomic columns, which are in accordance with the projection of 2*H*-NbSe_2_ along the [100] direction^22^ depicting that the Nb atoms are coordinated trigonal prismatically and not octahedrally by Se atoms. The atomic columns resolved in the PbSe layer agree with a projection of bulk PbSe along the [100] direction^46^.

(PbSe)_1.14_(NbSe_2_)_*n*_ ferecrystals are investigated as polycrystalline thin films of thickness 43.0(1) nm, 37.3(1) nm and 36.8(1) nm for *n* = 1, 2 and 3, respectively, as determined by X-ray reflectivity (XRR). To visually inspect a cross-section of the ferecrystals (PbSe)_1.14_(NbSe_2_)_*n*_, high-angle annular dark-field scanning transmission electron microscopy (HAADF-STEM) images were obtained for *n* = 1 and 3 (Fig. 1). The HAADF-STEM images reveal the structural properties typical for ferecrystals^15^,^16^,^19^: in-plane crystallinity, chemically and structurally abrupt interfaces and turbostratic disorder. The in-plane grain size ranges from a few to several tens of nanometers (see Supplementary Fig. S1). Some NbSe_2_ layers show a projection typical for a trigonal prismatic coordination of Nb by Se atoms (Fig. 1c), as found in analogous single crystalline bulk platelets, misfit layer compounds (MLCs)^20^,^21^ and in bulk 2*H*-NbSe_2_^22^. Due to the turbostratic disorder, a local octahedral coordination of the Nb atoms, as in bulk 4*H*-NbSe_2_ cannot be fully excluded, but projections typical for the octahedral coordination were not observed.

**Figure 2:**
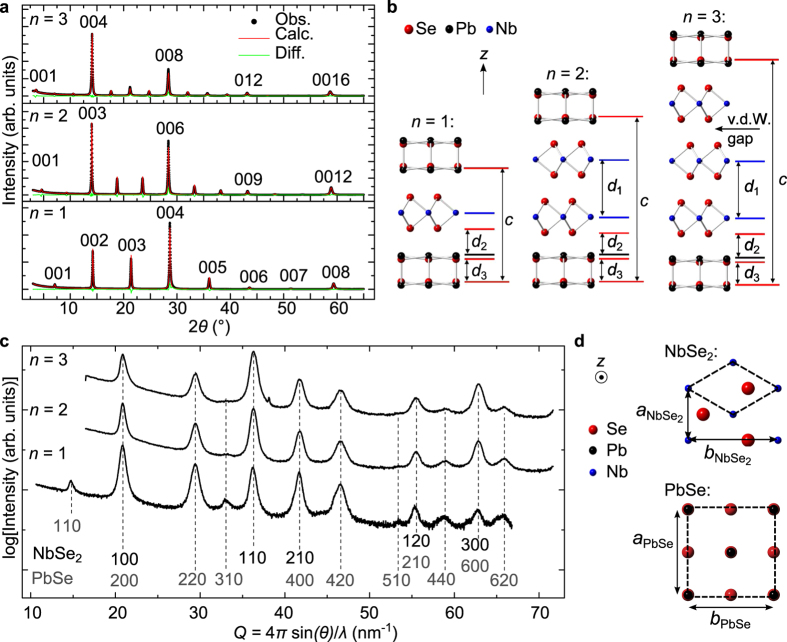
Crystal structure determination of the ferecrystals (PbSe)_1.14_(NbSe_2_)_*n*_ with *n* = 1, 2 and 3. (**a**) XRD scans (Obs.) showing the periodically layered structure of the ferecrystals. A Rietveld refinement (Calc.) was performed to determine atomic plane distances along the stacking direction. The difference (Diff.) between observed and calculated data is also shown. (**b**) Structure models of the (PbSe)_1.14_(NbSe_2_)_*n*_ ferecrystals. The repeat unit thickness *c* and the distances *d*_1_, *d*_2_ and *d*_3_ between atomic planes along the stacking direction are indicated. A van der Waals (v.d.W.) gap between the NbSe_2_ layers is present and is marked for *n* = 3 exemplarily. (**c**) In-plane XRD scans with peaks, which can be indexed to a hexagonal (NbSe_2_) and a square (PbSe) basal plane in accordance with the bulk structures. The peaks can be indexed as *hk*0 separately for NbSe_2_ (black) and PbSe (gray). Dashed lines indicate the peak positions. (**d**) Structure models of a PbSe and a NbSe_2_ projected onto the layer plane. Unit cells are indicated by dashed lines.

X-ray diffraction (XRD) scans of the ferecrystals are shown in Fig. 2. The observed peaks shown in Fig. 2a can be indexed as (00*l*) Bragg reflections, revealing the periodically layered structure of the ferecrystals. The repeat unit thicknesses *c* obtained from these XRD scans are in agreement with those reported for the corresponding MLCs (see Table 1), with a repeat unit comprising *n* NbSe_2_ monolayers and one atomic bilayer of PbSe, as depicted in Fig. 2b. The *c*-parameter increases linearly with *n* and the NbSe_2_ layer thicknesses are in agreement with NbSe_2_ monolayers in bulk NbSe_2_ (see Supplementary Fig. S2).

A van der Waals gap is present between two subsequent NbSe_2_ monolayers for ferecrystals with *n* = 2 and 3 (Fig. 2b). A Rietveld refinement of the atomic plane positions along the stacking direction has been performed (Fig. 2a). The obtained distances *d*_1_, *d*_2_ and *d*_3_, depicted in Fig. 2b, are listed in Table 1 and are similar to those reported for MLCs and bulk NbSe_2_, indicating that no interstitial atoms are present between the layers. Furthermore, the in-plane XRD scans of the ferecrystals show independent constituent structures that do not change as the thickness of the NbSe_2_ layer is increased, indicating there are no distortions that induce strain (see bold values in Table 1, Fig. 2c and Supplementary information, section 1). The MLCs, however, have a common in-plane *b* axis which distorts both constituent structures. The amount of the distortion changes as the NbSe_2_ thickness increases to balance the strain between the constituents and minimize the total energy. For MLCs, the area ratio between the PbSe and NbSe_2_ units has been determined as 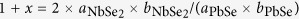 and the resulting misfit parameter *x* is listed in Table 1. For ferecrystals 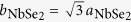 (hexagonal in-plane unit cell) and *b*_PbSe_ = *a*_PbSe_ (square in-plane unit cell) and *x* is similar or higher than in MLCs. So, while the ferecrystals studied here have stacking sequences identical to the bulk misfit layered compound single crystals studied previously^20^,^21^,^23^,^24^, they have distinctly different in-plane structures and orientations between the constituent layers. In particular, the NbSe_2_ layers in the ferecrystal have the same hexagonal symmetry as found in bulk NbSe_2_ and presumably in exfoliated single or few layer NbSe_2_ flakes.

### Normal state electrical properties

**Figure 3:**
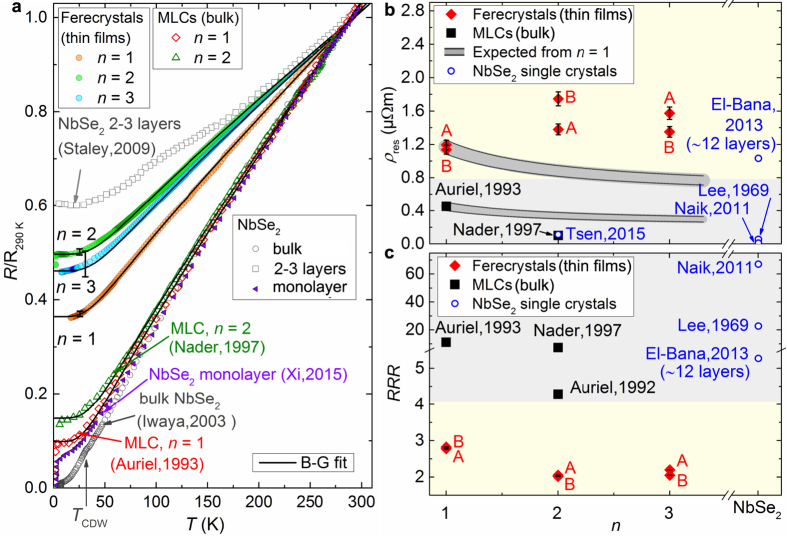
Normal state electrical properties of ferecrystals (PbSe)_1.14_(NbSe_2_)_*n*_. (**a**) Temperature-dependent in-plane electrical resistance normalized to the resistance at *T* = 290 K. The error bars for the resistances of the ferecrystals have been determined by the difference in resistance between the measurements of two samples and are shown exemplarily for one temperature. The data reported for NbSe_2_ bi-trilayers^12^ are from two-terminal measurements. The data for the ferecrystal thin films and for the bulk MLCs single crystals reported by Nader *et al*.^20^ and Auriel *et al*. 1993^23^, for an NbSe_2_ monolayer reported by Xi *et al*.^1^ and for bulk NbSe_2_ reported by Iwaya *et al*.^47^ are from four-terminal measurements. Black solid lines are Bloch-Grüneisen fits for ferecrystals and MLCs. The charge density wave transition temperature of bulk NbSe_2_ (*T*_CDW_) is indicated. (**b**) In-plane residual resistivity *ρ*_res_ in dependence on *n* for (PbSe)_1.14_(NbSe_2_)_*n*_ ferecrystal thin films and MLC bulk reported by Nader *et al*.^20^ and Auriel *et al*. 1993^23^, for NbSe_2_ bilayers reported by Tsen *et al*.^13^, for 12 layers of NbSe_2_ reported by El-Bana *et al*.^48^ and for bulk NbSe_2_ reported by Naik *et al*.^8^ and Lee *et al*.^30^. The labels A and B indicate a clover-leaf (A) and a cross-shaped sample (B), respectively. (**c**) Residual resistance ratio *RRR* of (PbSe)_1.14_(NbSe_2_)_*n*_ ferecrystals and data reported for bulk MLC single crystals (PbSe)_1+*x*_(NbSe_2_)_*n*_ reported by Auriel *et al*. 1993^23^ and Nader *et al*.^20^. The data reported by El-Bana *et al*.^48^ and Tsen *et al*.^13^ are data from exfoliated flakes. The data reported by Lee *et al*.^30^ and Naik *et al*.^8^ are data from bulk NbSe_2_ single crystals.

At room temperature (rt) the electrical resistivity values of the ferecrystals (PbSe)_1.14_(NbSe_2_)_*n*_ are *ρ*_rt_ = 3.3(3) μΩm for *n* = 1, 3.3(6) μΩm for *n* = 2 and 3.0(4) μΩm for *n* = 3. The temperature dependences of the in-plane resistances normalized to the resistances at *T* = 290 K are shown in Fig. 3a.

All samples show a metal-like temperature dependence of the resistance with a linear increase in the resistance for temperatures between *T* ≈ 50 K and 290 K. Differences in our electrical resistivity measurements between samples type A (clover-leaf shaped) and B (cross-shaped) are ascribed to cases where macroscopic defects such as scratches (holes) were present, which may influence the results of the van der Pauw technique. The *RRR* decreases with increasing *n* from *RRR* = 2.80(3) for *n* = 1 to *RRR* = 2.12(8) for *n* = 3 (Fig. 3c).

**Figure 4:**
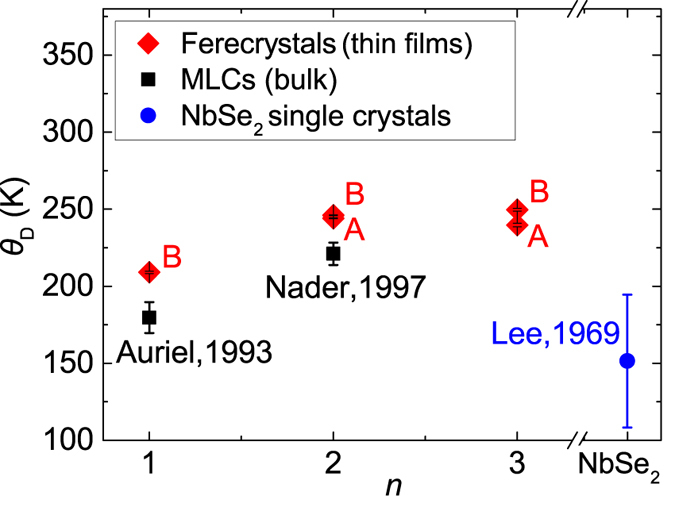
Debye temperatures of (PbSe) _1.14_(NbSe_2_)_*n*_ ferecrystals as a function of *n* in comparison to those of bulk (PbSe)_1+x_(NbSe_2_)_*n*_ MLC single crystals reported by Auriel *et al*.^23^ and Nader *et al*.^20^ and bulk NbSe_2_ reported by Lee *et al*.^30^ in comparison to those of bulk (PbSe)_1+x_(NbSe_2_)_*n*_ MLC single crystals reported by Auriel *et al*.^23^ and Nader *et al*.^20^ and bulk NbSe_2_ reported by Lee *et al*.^30^. Error bars for Debye temperatures have been obtained from the 95% confidence intervals of the regression using equation (1).

The temperature dependence of the resistance of the ferecrystals can be fitted by the Bloch-Grüneisen model


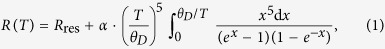


with the fit parameters *R*_res_, *a* and *θ*_*D*_, which are the residual resistance, a temperature-independent factor and the Debye temperature, respectively. Our fits are shown in Fig. 3a as black solid lines. The Debye temperatures obtained from the regression are displayed in Fig. 4 as a function of *n*. They increase from *θ*_*D*_ = 209(1) K for *n* = 1 to *θ*_*D*_ = 245(2) K for *n* = 2 and *θ*_*D*_ = 245(7) K for *n* = 3.

Hall coefficients *R*_H_ = *V*_H_ · *d/*(*B* · *I*) were measured for *n* = 1, 2 and 3 in the normal state at 10 K (see Supplementary Fig. S4(a,b)). The Hall coefficient *R*_H_ decreases systematically with increasing number *n* of NbSe_2_ layers with values ranging from 1.98(1) × 10^−3^ cm^3^/As for *n* = 1 to 1.085(6) × 10^−3^ cm^3^/As for *n* = 3. Carrier densities as calculated from a one-band model (*p* = 1/(e*R*_H_)) range from *p* ≈ 3 × 10^3^ cm^−3^ for *n* = 1 to *p* ≈ 5.5 × 10^3^ cm^−3^ for *n* = 3 (shown in the Supplementary Fig. S4(c)).

### Superconductivity in ferecrystals

**Figure 5:**
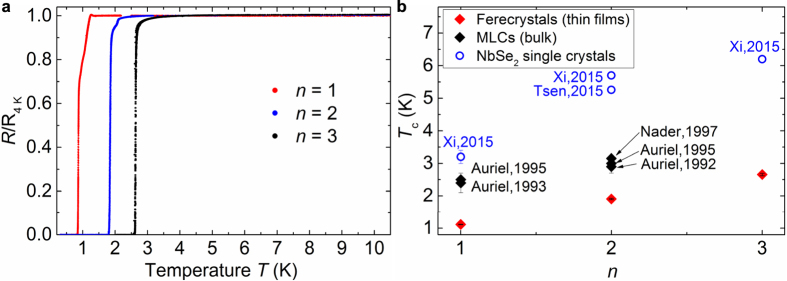
Transition temperatures to superconductivity. (**a**) Temperature-dependent resistances of (PbSe)_1.14_(NbSe_2_)_*n*_ ferecrystals measured in one of the van der Pauw measurement configurations, using the lock-in technique and normalized to the resistance at *T* = 4 K. (**b**) Superconducting transition temperatures *T*_*c*_ for (PbSe)_1.14_(NbSe_2_)_*n*_ ferecrystals, MLCs and NbSe_2_ single crystals. Data for bulk (PbSe)_1+*x*_(NbSe_2_)_*n*_ MLC single crystals are reported by Nader *et al*.^20^, Auriel *et al*. 1992^21^, Auriel *et al*. 1993^23^ and Auriel *et al*. 1995^29^. Reports on *T*_*c*_ by Oosawa *et al*. are for pressed pelletized MLCs^24^. For NbSe_2_ mono-, bi- and trilayers (*n* = 1, 2, 3) *T*_*c*_ is given as reported by Xi *et al*.^1^ and for bilayers it is reported by Tsen *et al*.^13^.

In search for superconductivity, we here investigated at low temperatures the normalized in-plane electrical resistivity of the ferecrystals (PbSe)_1.14_(NbSe_2_)_*n*_ with *n* = 1, 2 and 3: All drop to zero in the temperature range between *T* = 0.3 K and 3 K, as shown in Fig. 5a. This abrupt decrease to zero resistivity clearly marks a transition from a normal-conducting to a superconducting state. This leads to well-defined transition temperatures *T*_c_ for each *n*. The occurrence of superconductivity in ferecrystals as an emergent phenomenon is not dependent on variations in sample size or shape, which may play a role in the absolute resistance or resistance ratio in the normal state instead (see Supplementary Fig. S5). The transition temperatures *T*_c_ determined as the temperatures at which the resistance has dropped to 90% of the normal state resistance at *T* = 4 K, are *T*_c_ = 1.11(2) K, 1.91(3) K and 2.66(4) K for *n* = 1, 2 and 3, respectively.

## Discussion

To investigate the influence of turbostratic disorder on the electrical properties, the properties of the ferecrystal thin films are compared to those of analogous MLCs, which however exist only as bulk samples. The MLCs are synthesized as single crystalline bulk platelets with a total thickness of about 10 μm–50 μm^20^,^23^,^24^,^25^,^26^. In ferecrystals the in-plane grain size ranges from a few to several tens of nanometers, as observed in the HAADF-STEM images. As shown in Fig. 3b, the in-plane residual resistivity values of the ferecrystals are 3–17 times higher than those of the MLCs^20^,^23^. We attribute this to the polycrystallinity and turbostratic disorder of the ferecrystals, leading to a higher defect scattering rate at grain boundaries and the interfaces between constituent layers.

Surprisingly, the ferecrystal *n* = 1 shows a lower *ρ*_res_ than those with *n* = 2 and 3. This differs from the MLCs and is somewhat unexpected, because the sample with *n* = 1 contains a smaller volume percent of metallic NbSe_2_ layers than samples with *n* = 2 and 3. Assuming that the conductivity of the PbSe layers is so low that it can be neglected in comparison to the conductivity of the NbSe_2_ layers 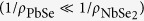, a simple parallel resistors model would give the following dependence of the in-plane resistivity on *n*, the number of NbSe_2_ layers:


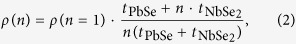


where *t*_Pbse_ = 6.06(2) Å and *t*_NbSe2_ = 6.36(1) Å are the thicknesses of a PbSe bilayer and a NbSe_2_ monolayer, respectively. Equation (2) is plotted in Fig. 3b for both ferecrystals and MLCs by scaling the *n* = 1 data (see Supplementary Fig. S2). The measured in-plane resistivity values for ferecrystals deviate from this simple model, suggesting the expected decrease in the resistivity with increasing NbSe_2_ content is superimposed by an effect that increases the resistivity. One possible explanation could be a decrease in grain size with increasing *n*, which results in an increase in resistivity, similar to what is observed for (SnSe)_1+*x*_(NbSe_2_)_*n*_ ferecrystals^27^. Our observation of a decrease in the residual resistance ratio *RRR* = *R*_290 K_/*R*_4 K_ with increasing *n* (Fig. 3c) supports this hypothesis.

While in general for the MLCs a qualitative decrease of the in-plane resistivity with increasing *n* is observed, the quantitative values also deviate from this simple model^20^,^23^. According to equation (2), for *n* = 2 a resistivity of about 76% of the resistivity of *n* = 1 is expected, however, for the MLC a stronger decrease to about 25% of the resistivity of *n* = 1 is measured. This might find its explanation in various sources: first, a decrease in interface scattering with increasing *n* due to the increasing thickness of the NbSe_2_ layers; second, a cross-over from a semimetal-like (single layer) to metallic behavior (bilayer) of NbSe_2_^9^,^28^; third, a higher electron-phonon scattering for the MLC with *n* = 1 compared to *n* = 2; and fourth, a change in the amount of charge transfer between the PbSe and the NbSe_2_ constituent as *n* is increased. A higher impurity or defect density in the MLC sample with *n* = 1 compared to *n* = 2 is unlikely, because the *RRR* of sample *n* = 1 is higher than for *n* = 2 (Fig. 3c). Therefore, we conclude that a simple parallel resistors model with 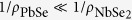 fails, both for MLCs and ferecrystals. In general, intrinsic origins for a deviation from the expected resistivity within a simple resistor model, both in ferecrystals as well as in MLCs, could be a change in carrier density with increasing *n* resulting from changing charge transfer between constituents as *n* is increased^14^. While the dependence of the band structure on *n* has not yet been reported, the determination of the charge carrier density within the one-band approximation yields increasing values for increasing *n* and hence supports the assumption of charge transfer. For the ferecrystals we find an increase from *p* ≈ 3 × 10^21^ cm^−3^ for *n* = 1 to *p* ≈ 5.5 × 10^21^ cm^−3^ for *n* = 3. For MLCs^29^ the value of the charge carrier density determined in such manner is identical for *n* = 1. This indicates that the amount of charge transfer for *n* = 1 due to a change in band structure is similar in both systems. For MLC of *n* = 2 or 3 the charge carrier densities are unknown.

The values of *θ*_*D*_ of the ferecrystals are above 200 K and are 20 K −30 K larger than those of the analogous MLCs^20^,^23^. All of them exceed the *θ*_*D*_ = 150 K of the NbSe_2_ single crystal^30^. Furthermore, Debye temperatures of about 200 K are typical for MLCs containing Nb*X*_2_^20^,^23^,^25^,^26^ and 240(3) K has been reported for the (SnSe)_1.16_(NbSe_2_) ferecrystal^31^. If the phonon spectra are dominated by the NbSe_2_ modes, then this suggests that the phonon spectra of ferecrystals and MLCs show only small differences, despite the structural distortions in the MLCs and the turbostratic disorder and decreased overall sample thickness of 40 nm of the ferecrystals.

In Fig. 5b we compare the superconducting transition temperatures *T*_*c*_ in dependence on the number *n* of NbSe_2_ layers in the repeat unit of the ferecrystals, their MLC analogues and NbSe_2_ mono-, bi- and trilayers. All of these transition temperatures were determined by resistance measurements with currents perpendicular to the crystallographic *c*-axis of NbSe_2_.

It is immediately evident that for all three systems, i.e. ferecrystal films, MLC bulk and individual NbSe_2_ single, bi- and trilayers, *T*_*c*_ increases monotonically with an increasing number *n* of NbSe_2_ layers (Fig. 5b). Furthermore, for each *n* the transition temperature *T*_*c*_ is highest for the ultra-thin NbSe_2_ film and lowest for the ferecrystals. In comparison, *T*_*c*_ for bulk NbSe_2_ has been reported in the range from 7.0 K to 7.4 K^1^,^32^,^33^,^34^,^35^, whereas bulk PbSe is reported to become superconducting only under high pressure^36^.

While the MLCs show a reduction to 50–80% of *T*_*c*_ of their ultra-thin film NbSe_2_ counterparts, the transition temperatures for the turbostratically disordered ferecrystals are further suppressed to 44% to 64% of *T*_*c*_ of the analogous crystalline MLCs, but superconductivity survives. This finding is remarkable as it shows that interlayer turbostratic disorder does not destroy superconductivity. The reduced superconducting transition temperature may arise from a reduction of the electron-phonon coupling and/or a change in the density of states at the Fermi level due to charge transfer between constituents, as both are key elements in the transition to superconductivity. The similar Debye temperatures of the ferecrystals and MLCs (Fig. 4) suggest similar phonon spectra, indicating that these are not the main cause of the strong differences in the *T*_*c*_ values. Such a strong reduction of *T*_*c*_ for the ferecrystals compared to the MLCs cannot be explained by the in-plane polycrystallinity of the NbSe_2_ layers either, as the transition temperature for polycrystalline NbSe_2_ has been reported to be identical^37^. A reduction of *T*_c_ by less than 2.5% due to polycrystallinity is reported for NbSe_2_ single crystals^37^,^38^. Furthermore, differences in the polytype of NbSe_2_ between ferecrystals and MLCs also appear unlikely. A detailed discussion is given in the Supplementary information, section 2.

The most probable origin for a reduction in *T*_*c*_ in ferecrystals as compared to MLCs is a difference in the amount of charge donation between constituents, *combined* with the turbostratic disorder caused by the misfit between the lattice constants of NbSe_2_ and PbSe. In the MLCs the NbSe_2_ and PbSe layers are aligned along one in-plane direction in which they are commensurate. In contrast, in ferecrystals the layers are not aligned and show independent lattice parameters. In spite of the rotational incoherence, the interface remains atomically abrupt. Charge transfer will occur until the chemical potential in both constituents is equal in both ferecrystals and MLCs, but the different structures in the polymorphs might change the magnitude of the transferred charge. In MLCs, the structural coherence between consecutive NbSe_2_ layers along the stacking direction may also lead to an enhanced coupling between the superconducting NbSe_2_ layers across the non-superconducting PbSe layers.

Unfortunately, we cannot determine unambiguously whether a change of charge transfer or a reduction of the electron-phonon coupling is the dominant factor in the decrease of *T*_c_. In both, the analysis of superconductivity for weak phonon-mediated coupling (BCS theory)^39^ as well as for strong coupling (McMillan’s formula)^40^, the gap equation links the critical temperature *T*_*c*_ in direct proportionality to the Debye temperature *θ*_*D*_ and as a function of the interaction strength *N*(0)*V*, where *N*(0) is the single-spin density of states at the Fermi level and *V* the electron-phonon coupling parameter. In our present study *N*(0) and *V* cannot be determined independently. Whether we consider the weak or strong coupling case in our analysis, using the experimental values for *T*_*c*_ and *θ*_*D*_ of the ferecrystals, MLCs or single layer NbSe_2_ we find an increase of interaction strength *N*(0)*V* with increasing number *n* of NbSe_2_ layers which correlates to an increasing (one-band) charge carrier density. On the one hand, an increase in charge carrier density is typically associated with an increase of density of states at the Fermi level. On the other hand, the increase of Debye temperature with increasing *n* indicates a change in phonon spectrum and therefore in the electron-phonon coupling. In order to confirm whether an increase in interaction strength is dominated by an increase in density of states or in electron-phonon coupling with increasing *n*, it is necessary to measure the density of states independently, for example by measuring a thermodynamic quantity such as specific heat or magnetization. However, for both cases of analysis we also find that consistently with the observed increase in *T*_c_ the interaction strength increases with increasing *n*. This is also the case for single- and multi-layer encapsulated NbSe_2_^11^.

The reports on individual exfoliated mono-, bi-, and trilayer flakes of NbSe_2_^1^,^13^ show higher *T*_*c*_ values than both ferecrystals and MLCs. A proximity effect in ferecrystals or MLCs could lead to superconductivity in the PbSe layers, leading to a lower total *T*_*c*_ of the ferecrystals and MLCs in comparison to individual NbSe_2_ layers. The proximity effect results in the lowering of *T*_*c*_ of a superconductor in contact with a normal conductor, resulting from the diffusion of charge carriers across the interface between the superconductor and the normal conductor^41^,^42^,^43^. Future investigations on doping of the PbSe layer (in analogy to previous studies^44^ on normal state properties) will be of importance in order to understand how a change in the charge carrier concentration directly affects the formation of superconductivity.

In summary, we have synthesized 2D layered materials of (PbSe)_1.14_(NbSe_2_)_*n*_ as turbostratically disordered ferecrystal thin films with *n* = 1, 2 and 3 using the MER method. The structural and electrical properties have been investigated and compared to identically stacked bulk crystalline MLC analogues. Both, ferecrystals and MLCs, show a metal-like temperature dependence of the resistivity and Debye temperatures in the range from 170 K up to 250 K, indicating similar phonon spectra. We demonstrate that, despite the structural orientation disorder, superconductivity occurs in the (PbSe)_1.14_(NbSe_2_)_*n*_ ferecrystals. In general, this opens the possibility of studying emergent phenomena, such as the two-dimensional superconductivity of individual NbSe_2_ layers, embedded in hybrid heterostructures of specifically designed stacking sequences. The observation of systematic variations in superconductivity in these compounds along with the ability to vary nanoarchitecture using the MER approach, provides an avenue to further our understanding of the interplay between neighbours in 2D heterostructures. While chemical substitutions have historically been used to probe structure-property relationships, the random potentials of isolated dopant atoms can compete with the interactions being probed. For example, the use of three component heterostructures of the form *ABCB* would enable the properties of the *C* layer to be probed as a function of carrier concentration via modulation doping from *A*. This type of investigation might also increase our understanding of the effect of substrates on the properties of 2D layers.

## Methods

The samples were synthesized applying the modulated elemental reactants (MER) method^15^,^16^,^17^, which includes physical vapor deposition of elemental layers onto silicon oxide on silicon substrates and subsequent annealing under a nitrogen atmosphere. The custom-built physical vapor deposition chamber achieves a base pressure of 6.7 × 10^−8^ mbar. Elemental niobium and lead sources (99.999% purity) were thermally evaporated using electron beam guns, while an effusion cell was used to thermally vaporize selenium (99.999% purity). Pneumatic shutters open for a preset duration to deposit elements in a desired scheme. Quartz crystal balances positioned over each source monitor the deposition rate. The binary pairs of elements must be deposited in a precise stoichiometric ratio, as determined by electron probe microanalysis with wavelength dispersive spectroscopy (EPMA-WDS). An iterative calibration process is used to determine the correct parameters for deposition. After deposition, the samples were annealed at 723 K for 60 minutes in a nitrogen environment (<1 ppm O_2_). The gentle annealing fulfills energetic requirements such that the amorphous elemental precursors self-assemble into the ferecrystalline form (see also Fig. S3). This synthesis method allows for the capture of samples in their kinetically favored, metastable state. The calibration of the ratio of the amounts of the two constituent layers corresponding to the misfit parameter *x* in (PbSe)_1+x_(NbSe_2_)_*n*_ is necessary. EPMA-WDS data were used to optimize deposition metrics, determine losses from sublimation during annealing, and examine the degree to which oxygen may have contaminated the sample.

Samples were prepared for high-angle annular dark-field scanning transmission electron microscopy (HAADF-STEM) using a focused ion beam (FEI Helios dual beam). An FEI Titan 80–300 operated at 300 kV was used to acquire HAADF-STEM images. The samples for HAADF-STEM and electrical characterization were from the same synthesis batches.

In order to measure the deposited layer thicknesses, to follow the structure as a function of annealing temperatures (Fig. S3) and annealing time and to determine the structure and crystallographic orientation, X-ray reflectivity and X-ray diffraction were applied using a Bruker D8 Discover XRD equipped with a Cu (0.154 nm) radiation source operated at 40 kV and 40 mA, a Göbel mirror and Bragg−Brentano optics geometry. Grazing incidence in-plane diffraction geometry was used to obtain in-plane diffraction patterns. The structural refinement along the *c*-direction was carried out using the FullProf program package^45^. An in-plane X-ray diffraction scan for sample (PbSe)_1.14_(NbSe_2_)_*n*_ with *n* = 1 was collected using a Rigaku Smartlab instrument with Cu radiation source. For samples with *n* = 2 and 3 in-plane X-ray diffraction scans were collected at the Beamline 33BM at the Advanced Photon Source at Argonne National Lab using an X-ray wavelength of 0.1127 nm.

The samples for the electrical characterization were synthesized on 300 nm silicon oxide on Si substrates using metal shadow masks, with a clover-leaf shape (samples A) and a cross shape (samples B) of size 4 mm × 4 mm. The samples were contacted at four points with 25 μm thin gold wires attached to the samples using indium (99.998% purity). The resistivity measurements were performed in an Oxford Instruments cryostat with a HelioxVL helium-3-insert. The resistances between room temperature and *T* = 2 K were measured during cooling and the temperature was measured using a calibrated Lakeshore Cernox thermometer located at the helium-3-pot. For the low-temperature resistance measurements between 300 mK and 45 K a calibrated Lakeshore ruthenium oxide thermometer, located below the chip carrier holding the sample, was used to determine the temperature. The in-plane resistivity at room temperature and at *T* = 4 K was determined using the van der Pauw method. For this purpose, a Keithley 6221 current source and a Keithley 2182 nanovoltmeter with direct currents of up to *I* = 50 μA were used to measure *I*-*V* curves. The total thicknesses *t* of the samples used for the calculation of the resistivity was determined from X-ray reflectivity scans obtained on other samples of the same synthesis batch. The in-plane resistance to determine the superconducting transition temperature was measured in one of the van der Pauw resistivity measurement configurations (four-terminal measurement) using a lock-in amplifier Signal Recovery DSP 7265 with *I*_rms_ = 2.5 μA. It was checked that the superconducting transition temperature was independent of current range in the current used for the determination of *T*_*c*_.

## Additional Information

**How to cite this article**: Grosse, C. *et al*. Superconducting ferecrystals: turbostratically disordered atomic-scale layered (PbSe)_1.14_(NbSe_2_)*_n_* thin films. *Sci. Rep.* **6**, 33457; doi: 10.1038/srep33457 (2016).

## Supplementary Material

Supplementary Information

## Figures and Tables

**Table 1:** Atomic plane distances and in-plane lattice constants of ferecrystals.

	*n* = 1		*n* = 2		*n* = 3		Bulk	
	FC film	MLC bulk	FC film	MLC bulk	FC film	MLC bulk	NbSe_2_ bulk	PbSe bulk
*c* (Å)	12.43(2)	12.47(8)^23^ 12.45(2)^24^	18.78(1)	18.76(1)^21^ 18.75(2)^24^	25.16(1)	25.087(6)^24^	—	—
*d*_1_ (Å)	—	—	6.179(1)	6.225(9)^21^	6.288(1)	Not rep.	6.27(2)^22^	—
*d*_2_ (Å)	2.929(1)	2.917(2)^23^	3.054(1)	2.89(2)^21^	3.042(1)	Not rep.	—	—
*d*_3_ (Å)	2.323(1)	2.56(2)^23^	2.764(1)	2.67(2)^21^	2.658(1)	Not rep.	—	—
*a*_NbSe2_ (Å)	**3.465(1)**	3.422(1)^23^ 3.456(3)^24^	**3.460(3)**	3.429(2)^21^ 3.449(2)^24^	**3.460(3)**	3.441(1)^24^	3.45(1)^22^	—
*b*_NbSe2_ (Å)	**6.002(1)**	6.056(1)^23^ 6.015(6)^24^	**5.993(6)**	6.011(2)^21^ 5.990(4)^24^	**5.993(6)**	5.986(2)^24^	5.98(2)^22^	—
*a*_PbSe_ (Å)	**6.031(1)**	6.202(3)^23^ 6.05(1)^24^	**6.033(2)**	6.143(4)^21^ 6.025(6)^24^	**6.029(5)**	6.011(4)^24^	—	6.121^46^
*b*_PbSe_ (Å)	**6.031(1)**	6.058(1)^23^ 6.013(7)^24^	**6.033(2)**	6.012(1)^21^ 5.991(3)^24^	**6.029(5)**	5.986(2)^24^	—	6.121^46^
*X*	0.14	0.10^23^ 0.14^24^	0.14	0.12^21^ 0.14^24^	0.14	0.14^24^	—	—

Repeat unit thickness *c*, atomic plane distances *d*_1_, *d*_2_ and *d*_3_, *a*- and *b*-parameters of NbSe_2_ and PbSe layers and misfit parameter *x* of (PbSe)_1+*x*_(NbSe_2_)_*n*_ ferecrystal thin films (FC) and bulk MLC single crystals^21^,^23^,^24^ obtained by XRD and respective values for bulk NbSe_2_ and PbSe^22^,^46^.
