# Supplementary material for: Superconducting ferecrystals: turbostratically disordered atomic-scale layered (PbSe)1.14(NbSe2)n thin films
Source: Sci Rep. 2016 Sep 16;6:33457. doi: 10.1038/srep33457 (PMC5025846; doi:10.1038/srep33457)
Supplement: Supplementary Information [file srep33457-s1.pdf]

Supplementary Information

Superconducting ferecrystals: turbostratically disordered atomic-scale layered $(PbSe)_{1.14}(NbSe_2)_n$ thin films

Corinna Grosse[1], Matti B. Alemayehu[2], Matthias Falmbigl[2], Anna Mogilatenko[1,3], Olivio Chiatti[1], David C. Johnson[2], and Saskia F. Fischer[1,*]

[1] Novel Materials Group, Humboldt-Universität zu Berlin, 12489 Berlin, Germany

[2] Department of Chemistry, University of Oregon, Eugene, Oregon 97403, United States

[3] Ferdinand-Braun-Institut, Leibniz-Institut für Höchstfrequenztechnik, 12489 Berlin, Germany

*E-mail: saskia.fischer@physik.hu-berlin.de

1. Structural characterization

1.1. Evaluation of high-angle annular dark-field transmission electron microscopy (HAADF-STEM) images

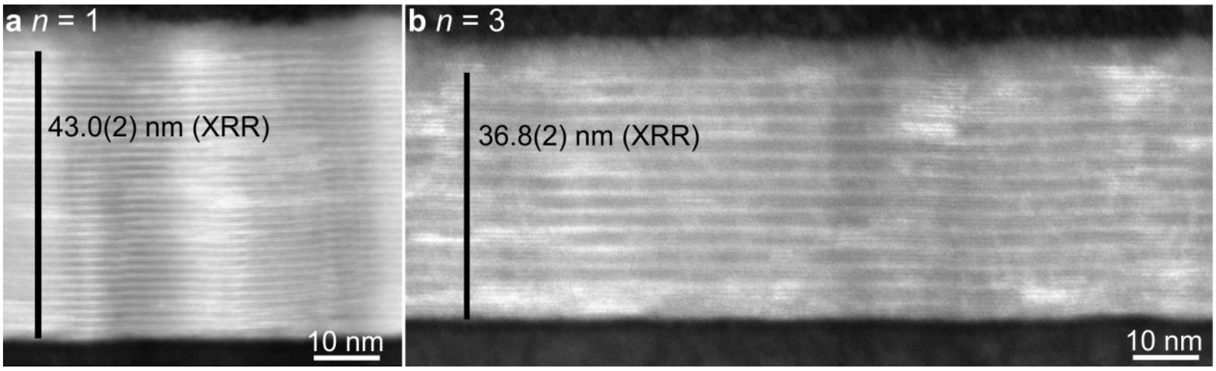

Supplementary Figure S1. HAADF-STEM overview images of ferecrystals $(PbSe)_{1.14}(NbSe_2)_n$ ferecrystal. (**a**) HAADF-STEM image for n = 1 and (**b**) for n =3. The total thickness determined by XRR is marked by a vertical black line.

Supplementary Figure S1 shows HAADF-STEM overview images of ferecrystals $(PbSe)_{1.14}(NbSe_2)_n$ with n = 1 and 3. Grain boundaries are visible. The grain size is estimated as 5 nm - 50 nm. The total film thickness agrees with the film thickness obtained by XRR.

1.2. Evaluation of cross-plane X-ray diffraction (XRD) scans

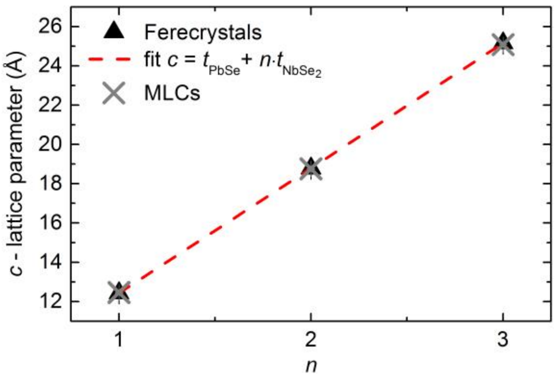

Supplementary Figure S2. Thickness of the repeat units. The repeat unit thicknesses of $(PbSe)_{1.14}(NbSe_2)_n$ ferecrystals and MLCs[4-6] have been determined by XRD. A linear fit obtained for the ferecrystals is shown.

As shown in Supplementary Figure 2, the *c*-lattice parameter increases linearly with *n* and a linear fit of *c*(*n*) yields an increase of the film thickness by $t_{\mathrm{NbSe_2}}$ = 6.36(1) Å for the addition of one $NbSe_2$ layer obtained from the slope and a thickness of t_{PbSe} = 6.06(2) Å of the PbSe bilayer, obtained as the intercept at $n = 0$. These thicknesses are roughly in agreement with those of $NbSe_2$ monolayers in bulk $NbSe_2$ which are 6.27(2) Å thick[1] and with atomic bilayers in bulk PbSe, which are reported 6.1213 Å or 6.117 Å thick[2,3]. Similarly, the fit of *c*(*n*) for the corresponding MLCs leads to $t_{\mathrm{NbSe_2}}$ = 6.323(5) Å and t_{PbSe} = 6.12(2) Å[4-6].

1.3. Evaluation of in-plane XRD scans

The in-plane XRD scans (main text, Figure 2c) contain peaks, which can be indexed as Bragg reflections (*hk*0) from individual $NbSe_2$ and PbSe layers assuming crystal structures equal to the respective bulk structures of $NbSe_2$ (hexagonal in-plane unit cell) and PbSe (square in–plane unit cell), with the *c*-axes parallel to the stacking direction in the ferecrystals. Structure models of a monolayer $NbSe_2$ and an atomic bilayer of PbSe projected onto the layer plane are shown schematically in Figure 2d. The resulting *a*- and *b*-parameters for ferecrystals and MLCs are listed in the main text Table 1. For ferecrystals, bulk $NbSe_2$ and bulk PbSe $b_{\mathrm{NbSe_2}} = \sqrt{3}a_{\mathrm{NbSe_2}}$ (hexagonal in-plane unit cell) and $b_{\mathrm{PbSe}} = a_{\mathrm{PbSe}}$ (square in-plane unit cell). In contrast, in MLCs the crystal structures distort in order to achieve lattice matching along the *b*-axes. Therefore, the $NbSe_2$ and PbSe layers in MLCs show rectangular in-plane unit cells[7,8].

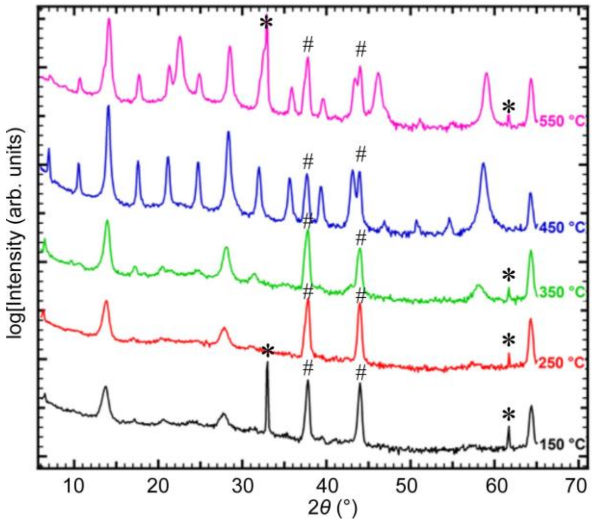

Supplementary Figure S3. X-ray diffraction data as a function of annealing temperature for $(PbSe)_{1.14}(NbSe_2)_3$ ferecrystal. The optimum annealing temperature was found to be 450 °C. Silicon substrate peaks are indicated by (*), aluminium stage peaks are present are indicated by (#).

2. Hall measurements

Supplementary Fig. S4(a) shows the magnetic field dependence of the Hall voltages V_H of the $(PbSe)_{1.14}(NbSe_2)_n$ ferecrystals at $T = 10$ K, multiplied by the total sample thickness t. The inset shows a schematic of the measurement setup. The measurements were performed using a lock-in amplifier (DSP 7265) with currents $I_{rms} = 5$ μA. The current I was applied between two contacts at two opposite arms of the cross-shaped sample and the voltage V_m was measured between the contacts at the other two arms during slow (0.2 T/min) increasing or decreasing of the magnetic field. Due to slightly offset positions of the contacts at the samples, for $B = 0$ a non-zero voltage V_m is measured. The Hall voltages have been determined by $V_H = V_m(B)-V_m(B = 0)$. The Hall voltages are linear in B, indicating that only one type of charge carrier dominates the electrical transport.

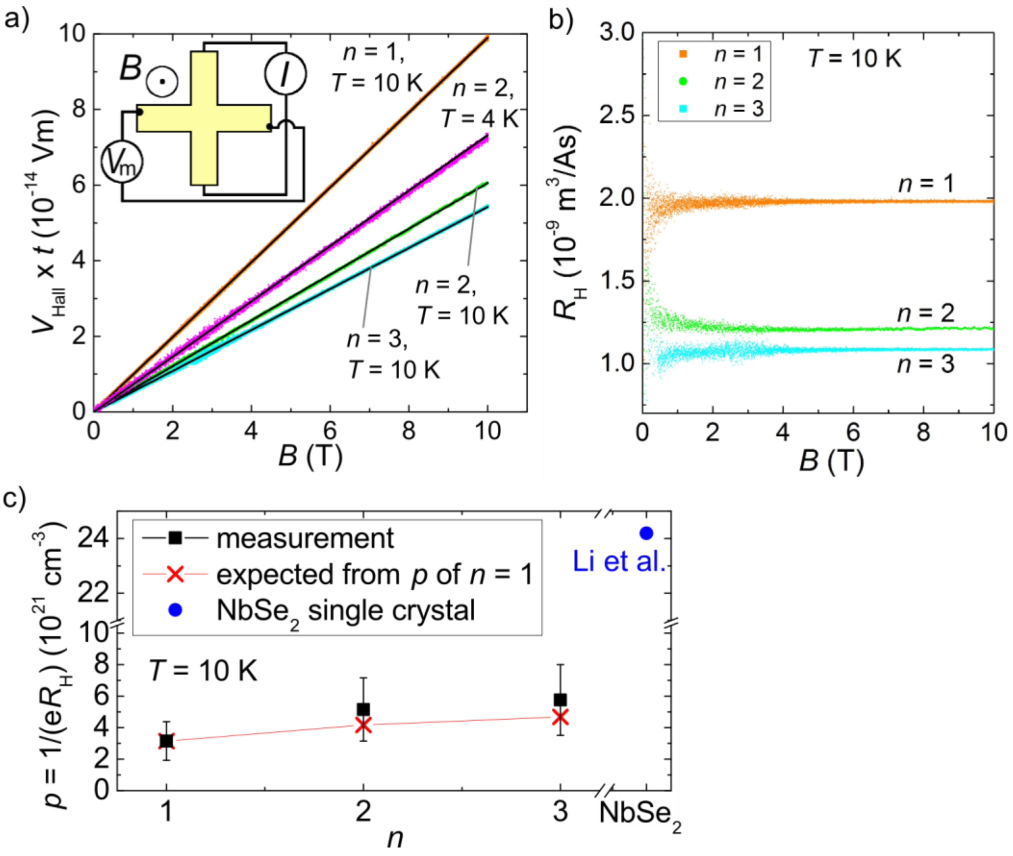

Supplementary Figure S4. (a) Hall voltage $V_H = V_m(B)-V_m(0)$ multiplied by thickness t and (b) Hall coefficients $R_H = V_H \cdot d/(B\cdot I)$ of the $(PbSe)_{1.14}(NbSe_2)_n$ ferecrystals for $T = 10$ K. An additional B–independent error of 0.39 x R_H due to the contact size has to be considered when calculating R_H. (c) Hole density p of the ferecrystals in comparison to values expected if the PbSe layers did not contribute to transport and the carrier density of the $NbSe_2$ layers were independent of n. The value for a bulk $NbSe_2$ single crystal reported by Li, *et al.*[9] is given by comparison.

3. Superconductivity

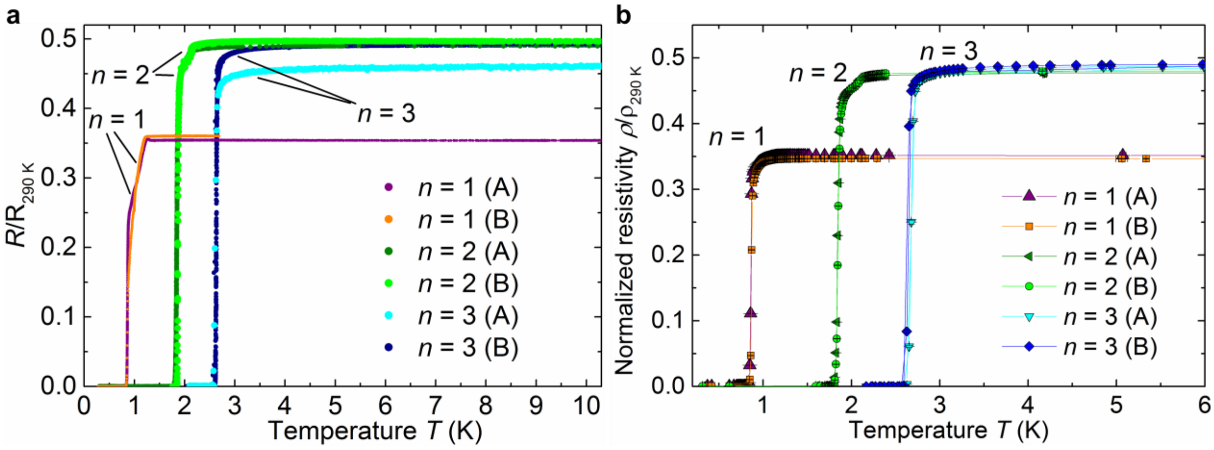

Supplementary Figure S5. Temperature-dependent resistances of $(PbSe)_{1.14}(NbSe_2)_n$ ferecrystals (**a**) Temperature-dependent resistances of $(PbSe)_{1.14}(NbSe_2)_n$ ferecrystals measured using the lock-in technique. (**b**) Temperature-dependent resistivity of $(PbSe)_{1.14}(NbSe_2)_n$ ferecrystals measured using the van der Pauw technique. The labels A and B indicate the clover-leaf (A) and cross (B) shaped samples.

A macroscopic difference between the MLC and the ferecrystal samples compared in Figure 5b of the main text is their total film thickness. The total thickness of the ferecrystals is about 40 nm, thereof 20 nm of $NbSe_2$ layers for n = 1, whereas the total thickness of the analogous MLCs is at least several micrometers[4,5,10]. However, the film thickness dependence of the transition temperature of 2*H*-$NbSe_2$ suggests that this should not play a major role: T_c of a 10 nm thin $NbSe_2$ flake is reported to be 5.7 K to 6.7 K[11,12] almost reaching the bulk value. Instead, the transition temperatures of the MLCs and ferecrystals are much lower than this, indicating that the total film thickness even in the case of superconductive coupling between the $NbSe_2$ layers does not predominantly determine T_c.

Possible reasons for the differences in T_c of the ferecrystals in comparison to the MLCs are differences in the polytype and stoichiometry of their $NbSe_2$ layers. However, the HAADF-STEM images of the ferecrystals show a trigonal prismatic coordination of the Nb atoms by Se atoms in accordance with the coordination reported for $NbSe_2$ layers in MLCs $(PbSe)_{1+x}(NbSe_2)_n$ and in 2*H*-$NbSe_2$, the most common polytype of $NbSe_2$[4,5,10,13,14]. In contrast, for the $NbSe_2$ layers in the ferecrystals $(SnSe)_{1+\gamma}(NbSe_2)_n$ indications for a mixture of a trigonal prismatic and an octahedral coordination have been reported[15]. $NbSe_2$ polytypes containing a mixture of trigonal prismatic and octahedral coordination, e.g. 4*H*-$NbSe_2$, have been reported to show a lower transition temperature to superconductivity ($T_c \leq 6.5$ K[16,17]) than the polytype 2*H*-$NbSe_2$ (T_c = 7 K[16-18]), which contains only trigonal prismatic coordination. Deviations from T_c = 7 K in $NbSe_2$ are also reported to be in connection with a non-stoichiometry, due to interstitial Nb atoms in $NbSe_2$, i.e. $Nb_{1+y}Se_2$, and T_c is reported to decrease from 7 K for $y = 0$ to approximately 5.5 K for $y = 0.02$[13,16]. However, there were no indications for interstitial atoms in the gaps between the ferecrystal layers in the HAADF-STEM images and in the XRD-scans obtained in this work. Therefore, a deviation in polytype or non-stoichiometry does not seem to be a probable explanation for the lower T_c of the ferecrystals in comparison to the MLCs.

Supplementary references

1. Brown, B. E. & Beerntsen, D. J. Layer structure polytypism among niobium and tantalum selenides. *Acta Cryst.* **18,** 31-36 (1965).

2. Noda, Y. *et al.* Temperature dependence of atomic thermal parameters of lead chalcogenides, PbS, PbSe and PbTe. *Acta Cryst. C* **43,** 1443-1445 (1987).

3. Mariano, A. N. & Chopra, K. L. Polymorphism in some IV-VI compounds induced by high pressure and thin-film expitaxial growth. *Appl. Phys. Lett.* **10,** 282(1967).

4. Nader, A., Recherches, C. D., Fourier, U. J. & Earth, R. Superconductivity in the misfit layer compound $(PbSe)_{1.12}(NbSe_2)_2$. *Solid State Comm.* **102,** 401-403 (1997).

5. Auriel, C., Roesky, R., Meerschaut, A. & Rouxel, J. Structure determination and electrical properties of a new misfit layered selenide $[(PbSe)_{1.10}NbSe_2]$. *Mat. Res. Bull.* **28,** 247-254 (1993).

6. Oosawa, Y., Gotoh, Y., Akimoto, J., Tsunoda, T., Sohma, M. & Onoda, M. Three types of ternary selenides with layered composite crystal structures formed in the Pb-Nb-Se system. *Japn. J. Appl. Phys.* **31,** 1096-1099 (1992).

7. Wiegers, G. A. Misfit layer compounds: Structures and physical properties. *Prog. Solid State Ch.* **24,** 1-139 (1996).

8. Falmbigl, M., Alemayehu, M. B., Merrill, D. R., Beekman, M & Johnson, D. C. In-plane structure of ferecrystalline compounds. *Cryst. Res. Technol.* **50,** 464-472 (2015).

9. Li, L., Xu, Z., Shen, J., Qiu, L. & Gan, Z., The effect of a charge-density wave transition on the transport properties of 2*H*-$NbSe_2$. *J. Phys.:Condens. Matter* **17,** 493–498 (2005).

10. Auriel, C., Meerschaut, A., Roesky, R. & Rouxel, J. Crystal structure determination and transport properties of a new misfit layer compound $(PbSe)_{1.12}(NbSe_2)_2$: "$PbNb_2Se_5$". *Eur. J. Solid State Inorg. Chem.* **29,** 1079-1091 (1992).

11. Staley, N. E., Wu, J., Elklund, P. & Liu, Y. Electric field effect on superconductivity in atomically thin flakes of $NbSe_2$. *Phys. Rev. B* **80,** 184505 (2009).

12. El-Bana, M. S., Wolverson, D., Russo, S., Balakrishnan, G., Paul, D. M. & Bending, S. J. Superconductivity in two-dimensional $NbSe_2$ field effect transistors. *Supercond. Sci. Technol.* **26,** 125020 (2013).

13. Auriel, C. *et al.* Electrical transport properties of mono-and bilayers misfit compounds $(MX)_{1+x}(TX_2)_m$, M = Sn, Pb; T = Ti, Nb; X = S, Se. *Eur. J. Solid State Inorg. Chem.* **32,** 947-962 (1995).

14. Monceau, P., Chen, J., Laborde, O., Briggs, A., Auriel, C., Roesky, R., Meerschaut, A. & Rouxel, J. Anisotropy of the superconducting properties of misfit layer compounds $(MX)_n(NbX_2)_m$. *Physica B* **194-196,** 2361-2362, (1994).

15. Alemayehu, M. B., Falmbigl, M., Ta, K. & Johnson, D. C. effect of local structure on the transport properties of $[(SnSe)_{1.16}]_1(NbSe_2)_n$ ferecrystals. *Chem. Mater.* **27,** 2158-2164 (2015).

16. Revolinsky, E., Spiering, G. A. & Beerntsen, D. J. Superconductivity in the niobium-selenium system. *Phys. Chem. Solids* **26,** 1029-1034 (1965).

17. Naik, I. & Rastogi, A. K. Charge density wave and superconductivity in 2*H*- and 4*H*-$NbSe_2$: A revisit. *Pramana* **76,** 957-963 (2011).

18. Nader, A. & Monceau, P. Critical field of 2*H*-$NbSe_2$ down to 50 mK. *SpringerPlus* **3,** 16 (2014).
